# Endoscopic retrograde cholangiopancreatography for the diagnosis and treatment of biliary *Fasciolopsis buski*: A rare case report and literature review

**DOI:** 10.1097/MD.0000000000042829

**Published:** 2025-06-20

**Authors:** Qiao Zhou, Yu Cao, Youcai Lv, Qiuling Zhao, Yating Wei, Yunlan Gu, Juanjuan Yang, Luxi Yang, Hongping Li

**Affiliations:** a Department of Gastroenterology, Digestive Disease Hospital, The Affiliated Hospital of Zunyi Medical University, Zunyi, China; b General surgery department, Digestive Disease Hospital, The Affiliated Hospital of Zunyi Medical University, Zunyi, China; c Department of Gastroenterology, Zhenning County People’s Hospital, Anshun, China.

**Keywords:** case report, common bile duct, endoscopic retrograde cholangiopancreatography, *Fasciolopsis buski*

## Abstract

**Rationale:**

*Fasciolopsis buski* can cause fasciolopsiasis, which usually parasitizes the duodenum and jejunum in humans, the biliary tract is rarely reported. However, as most infections are asymptomatic or present with nonspecific symptoms, they are often not diagnosed or sometimes even misdiagnosed.

**Patient concerns:**

We report the rare case of a 59-year-old male who was admitted to our hospital with a history of recurrent and unexplained abdominal pain for ≈11 years and worsening symptoms for 5 days.

**Diagnoses:**

Following laboratory tests, magnetic resonance cholangiopancreatography, endoscopic retrograde cholangiopancreatography and pathological biopsy, the final diagnosis was *F buski* in the common bile duct.

**Interventions:**

After excluding contraindications to surgery, an adult worm was successfully extracted using endoscopic retrograde cholangiopancreatography.

**Outcomes:**

No adverse reactions were noted. After 1 year of follow-up, the symptoms of abdominal pain had improved, which confirmed the diagnosis and showed a significant therapeutic effect.

**Lessons:**

This is the first report of the treatment of *F buski* in the common bile duct using endoscopic retrograde cholangiopancreatography. A review of the literature on *F buski* in humans was conducted to summarize its clinical features and treatment, providing a valuable therapeutic technique for clinicians.

## 1. Introduction

*Fasciolopsis buski*, the largest giant intestinal trematode, belongs to the family Hepatidae, and was first discovered in the duodenum of an Indian sailor during an autopsy by George Busk in 1843.^[1]^ This parasite has been reported in several countries, including Bangladesh, India, Indonesia, Malaysia, Vietnam, Laos, Thailand, and China. It is estimated that ≈10 million people worldwide are infected with this parasite.^[2]^ However, with the development of society and the economy, the incidence of this parasitic infection has significantly decreased and it has become relatively rare in clinical practice. Reports of this parasite are usually documented in the duodenum and jejunum of humans and are rarely reported in the common bile duct. The novelty of this paper lies in the rare occurrence of *F buski* in the biliary tract. The doctors successfully removed the *F buski* through endoscopic retrograde cholangiopancreatography (ERCP). The paper emphasizes the importance of using minimally invasive procedures to accurately diagnose and treat parasitic diseases of the digestive system, thereby reducing the risk of misdiagnosis, missed diagnosis, or the greater harm that may be caused to patients by open abdominal surgery.

## 2. Case presentation

A 59-year-old male experienced intermittent epigastric pain for ≈11 years. Because the epigastric pain had aggravated for 5 days, the patient sought medical care at the First People’s Hospital of Zunyi, where abdominal computed tomography (CT) revealed dilatation of the intra- and extra-hepatic bile ducts. Laboratory tests showed a leukocyte 7.7×10^9^/L, eosinophil ratio of 5.3%, absolute eosinophil value of 0.41×10^9^/L, hemoglobin level of 161 g/L, platelets of 192×10^9^/L. The patient was admitted to our hospital for further diagnosis and treatment. There was no significant past medical history. Physical examination revealed the patient had mild tenderness in the upper abdomen. Laboratory tests showed a leukocyte 6.67×10^9^/L, eosinophil percentage of 0.1, absolute eosinophil value of 0.68×10^9^/L, hemoglobin level of 145 g/L, platelets of 142×10^9^/L. The levels of the biomarkers were unremarkable.

**Figure 1.**
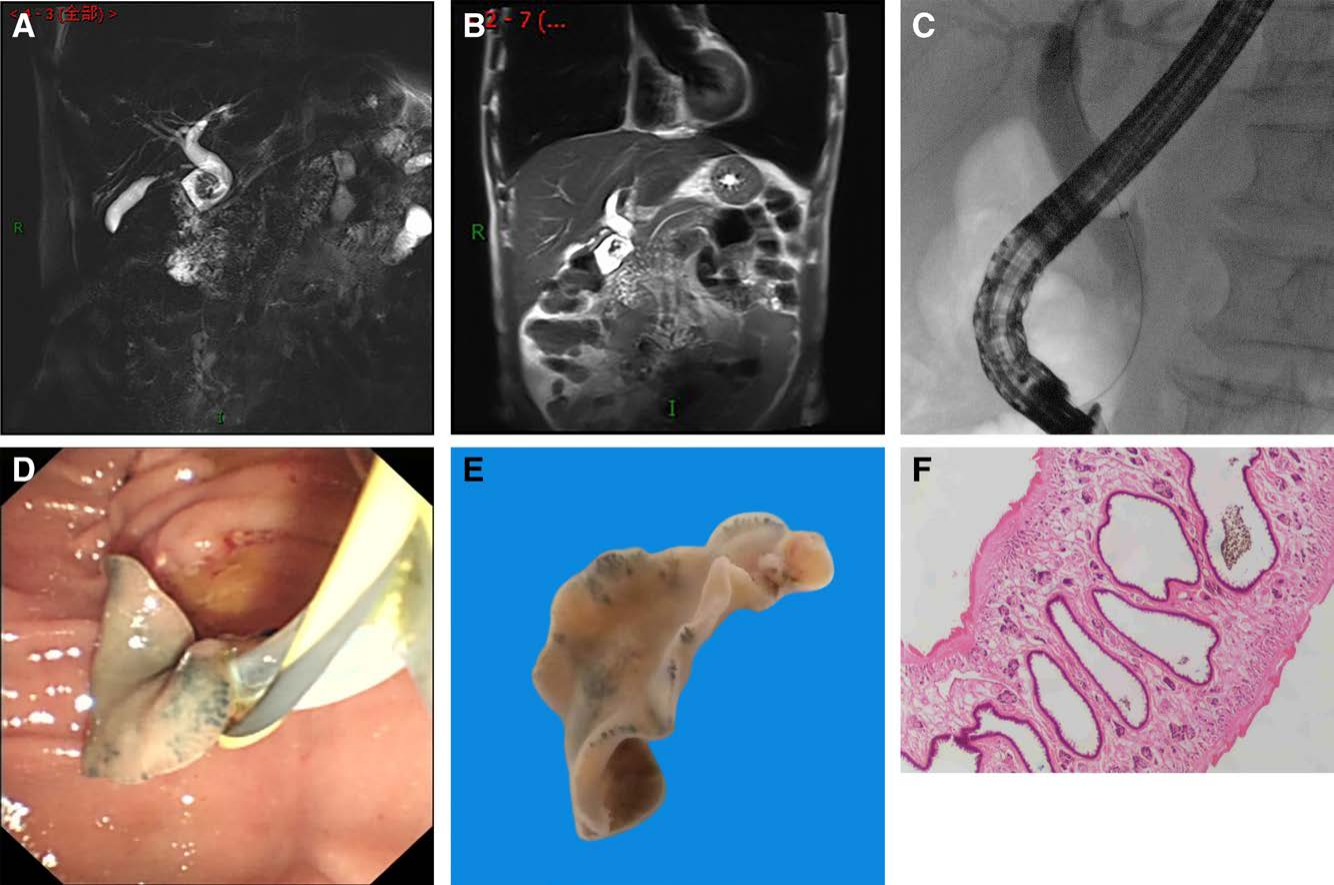
(A and B) MRCP showing lower choledochal stenosis and dilatation of intra- and extra-hepatic bile ducts. (C) ERCP intraoperative contrast showing dilatation of the choledochal ducts and a striated filling defect in the lower segment. (D) Choledochal worm extraction under ERCP. (E) A live adult *F buski*; F: histopathological picture of *F buski.* ERCP = endoscopic retrograde cholangiopancreatography, MRCP = magnetic resonance cholangiopancreatography.

To clarify the etiology of the abdominal pain, magnetic resonance cholangiopancreatography was performed: stenosis of the lower end of the common bile duct, dilatation of the intra- and extra-hepatic bile ducts, and tortuous streak-like shadows in the lumen of the common bile duct (Fig. 1A and 1B). Upon further inquiry into the patient’s medical history and lifestyle, it was revealed that the patient had a preference for consuming raw pig blood and had been living in close proximity to a river for an extended period. Given that Guizhou province used to be an epidemic area for parasitic diseases, although the incidence rate is very low at present, it cannot be ignored. Therefore, abdominal pain cannot be excluded from the diagnosis of biliary parasitic diseases. After excluding patients with contraindications for surgery, ERCP was performed. A TJF-260 electronic duodenoscope was inserted into the descending part of the duodenum, where a duodenal diverticulum was observed, with the papilla located below the diverticulum. Under the guidance of a 0.035-Fr yellow zebra guidewire, a 3-lumen papillotome was used to selectively cannulate the bile duct. After decompression, cholangiography was performed by injecting ≈20 mL of 30% iohexol. X-ray imaging revealed no visualization of the gallbladder, mild dilatation of the common bile duct with a striated filling defect in the lower segment (Fig. 1C), A 0.4-cm incision was made in the sphincter of Oddi using a crescent-shaped sphincterotome, and a stone retrieval balloon was used to probe the filling defect, which could not rule out the presence of a parasite. Subsequently, a live parasite was retrieved using a stone basket (Fig. 1D). The parasite measured ≈2.4 × 1.1 × 0.2 cm, the anterior end of the parasite featured a sucker, with undulating margins, and the posterior end had a tail-like structure (Fig. 1E). No other parasites were detected in the biliary tract. Nasal bile duct drainage was then performed. The patient was asked to fast temporarily, and the amylase level was slightly elevated on repeat examinations. Abdominal CT revealed no significant biliary ductal dilation on the second postoperative day, after which the nasal bile duct was removed. The parasite was sent for pathological biopsy, which showed that it was an adult worm of *F buski* (Fig. 1F), and contacted the Zunyi City Center for Disease Control for treatment.

During the admission workup, the patient exhibited no significant abnormalities in inflammatory markers or liver function tests. There was no fever, jaundice, or severe abdominal pain, and Murphy sign was negative, leading us to exclude cholecystitis. CT scans showed no low-density cystic lesions or low-density masses within the bile ducts, most importantly, ERCP and histopathological biopsy findings did not support the diagnosis of biliary cysts or gallbladder cancer. The patient had a history of consuming raw pig blood and living near a river and presented with elevated eosinophil counts, all of which raised suspicion for a parasitic infection. Eventually, ERCP uncovered the long-hidden parasite. The pathological results were surprising: it was identified as *F buski*, a rare occurrence in the biliary tract. The patient received 15 mg/kg of praziquantel via oral deworming. After 1 year of follow-up through telephone interviews, the symptoms of abdominal pain had improved, which confirmed the diagnosis and showed a significant therapeutic effect.

## 3. Discussion

*F buski* uses specific snail species as intermediate hosts; however, humans and pigs are its final hosts.^[2]^ The eggs of *F buski* are excreted in feces, hatched in an aquatic environment, and transformed into cercaria, which penetrates the intermediate host and promotes its development into cercaria. Then these cercariae encyst on aquatic plants, forming metacercariae. After ingesting the final host, the metacercariae shed in the duodenum, and the larvae adhered to the mucosal layer of the duodenum or proximal jejunum. After 1 to 3 months of maturity, they evolve into adults, relying on the intestinal blood and mucus of the host for survival. These adult parasites continue to grow and ovulate, initiating a new round of parasite development.^[3]^

Infection in humans is predominantly acquired through the ingestion of water or aquatic vegetation contaminated with metacercarial cysts, such as those found in bamboo shoots, chestnuts, and caltrops, as well as through the consumption of pork.^[2]^ The duodenum and initial portion of the jejunum were the most frequently parasitized regions. In cases of mild infection, symptoms may be absent. The suckers firmly attach to the mucosal layer, resulting in localized mucosal necrosis, desquamation, inflammatory responses, hemorrhage, edema, and in more severe cases, formation of abscesses or ulcerations.^[4]^ In cases of severe infection, the parasites may adhere to diverse regions of the gastrointestinal tract, including the stomach, ileum, cecum, appendix, and potentially the biliary system, precipitating a range of complications such as gastrointestinal bleeding,^[5]^ intestinal ulceration,^[6]^ intestinal perforation,^[7]^ intestinal obstruction,^[8]^ acute appendicitis,^[9]^ and biliary disorders.^[10]^ Clinical manifestations include nausea, vomiting, vomiting of the blood, melena, abdominal pain, and diarrhea.^[11]^

A case reported by Wang et al^[8]^ involved a patient who exhibited complete intestinal obstruction characterized by the absence of bowel movements and flatus, along with nausea and vomiting. During endoscopic examination, an unprecedented number of *F buskis* (153) were removed. Persistent adherence of these parasites to the intestinal mucosa can precipitate localized abscesses, which may advance to ulceration. Failure to detect and treat this condition in a timely manner can result in intestinal perforations. As illustrated in the case reported by Bhattacharjee et al,^[7]^ a 10-year-old boy in India underwent exploratory laparotomy for abdominal pain, which revealed ileal perforation with purulent ascites and numerous viable adult worms in the peritoneal cavity. There are also cases reported that *F buski* adsorbed in the ileocecal region,^[9]^ then moved to the appendiceal orifice resulting in blockage of the appendiceal orifice, increased pressure in the lumen, resulting in the symptom of metastatic right lower abdominal pain, inflammation and abscess in some areas, and appendiceal swelling observed under ultrasound or CT, these manifestations confuse clinicians and are easily misdiagnosed as acute appendicitis, and may even be subjected to laparoscopic or even open surgical resection of appendix,^[12]^ representing an instance of overmedicalization due to diagnostic error. This report describes an exceedingly rare case of *F buski* infection of the biliary tract. The parasites penetrate the intestinal wall from the small intestine, traverse the peritoneal cavity, and subsequently infiltrate the liver and bile ducts, resulting in upper abdominal pain and symptoms such as fever and jaundice. The adherence of these flukes to the walls of the biliary ducts can be radiographically misinterpreted as signs of cholecystitis, biliary cysts, or neoplastic conditions, potentially prompting invasive procedures such as laparoscopic or open cholecystectomy and exploration of the common bile duct.^[10,13]^ Such misdiagnoses can impose a burdensome clinical course on patients.

Furthermore, infection is associated with a compromised immune response and the potential for acute renal impairment,^[14]^ presenting as diminished appetite, weight loss, malnutrition, anemia, or oliguria. Beyond these clinical signs, the systemic absorption of noxious and allergenic metabolites excreted by parasites can induce systemic toxicity and allergic manifestations, including ascites and generalized and facial edema.^[15]^ In extreme cases, it may progress to shock or fatal outcomes.^[16]^

A review of related cases of fasciolopsiasis over the past decade (Table 1) revealed a predominance of reports from India and China, underscoring the enduring link between economic conditions and health infrastructure development. Affected individuals present with pronounced gastrointestinal symptoms that lack specificity. The gastrointestinal tract is the most frequent site of parasitism, and detection of *F buski* is often achieved through gastroenteroscopy.^[20,21]^ Infection can also be confirmed by the identification of adult worms and eggs in vomitus or fecal samples.^[22]^ During clinical work, upon encountering patients with suspected fasciolopsiasis, it is necessary to improve the routine stool examination to detect the presence of adult worms and eggs and the presence of unexplained eosinophils in routine blood examination, and further improve the enzyme-linked immunosorbent assay of *F buski* adult antigen.^[23]^

**Table 1:** Clinical features and treatment of fasciolopsiasis in the past decade.

Author	Year	Country	Age/sex	Life habits	Symptoms	(Preoperative) Diagnosis	Location	Quantity	Vomitus/stool examination	Treatment	Outcome
Deka et al^[17]^	2022	India	4/F	Catching fish	Abdominal pain and diarrhea	Severe malnutrition	Gastrointestinal tract	Mass	Yes	Praziquantel, nutritional support	Recovered
Wu et al^[10]^	2020	China	15/F	–	Abdominal pain	Choledochal cysts	Common bile duct	1	No	Exploratory laparotomy, praziquantel	Recovered
Jha et al^[6]^	2019	India	40/F	Eating of chestnuts	Abdominal pain, vomiting	Intestinal parasite	Stomach, duodenum	Plural	Yes	Gastroscopy, praziquantel	Recovered
Sah et al^[18]^	2019	Nepal	22/W	Eating of raw aquatic crops	Abdominal pain, distension	Portal hypertensive gastropathy	Duodenum	1	Yes	Gastroscopy, praziquantel	Recovered
Sarma et al^[19]^	2015	India	8/F	Eating of chestnuts, pond water	Vomiting blood	Upper gastrointestinal bleeding	Duodenum	1	Yes	Gastroscopy, praziquantel	Recovered
Cao et al^[9]^	2015	China	45/F	–	Abdominal pain	Acute appendicitis	Ileocecal region	2	Yes	Enteroscopy, praziquantel	Recovered
Cao et al^[9]^	2015	China	71/F	–	Abdominal pain and diarrhea	Acute appendicitis	Ileocecal region	3	No	Enteroscopy, praziquantel	Recovered

F = female, M = male, no = no adult worms and eggs in vomitus or stool, yes = finding adult worms and eggs in vomitus or stool, – = not described.

In addition to routine imaging, gastroscopy and ERCP are diagnostic methods for fasciolopsiasis. In the current environment where minimally invasive development is extremely promising, ERCP is an important method for the diagnosis of biliopancreatic diseases. It is particularly prominent in the treatment of parasitic cholangitis caused by parasites,^[24]^ especially in the decompression of parasitic biliary obstruction, and is the preferred treatment plan for parasitic diseases of the bile ducts such as hepatic hydatidosis, ascaris, and fascioliasis. During ERCP, the morphology of the parasites can be directly visualized, and the contours of the biliary ducts, as well as sites of stenosis or dilation, can be clearly delineated. In addition, biliary obstructions and inflammatory lesions caused by the parasites are also visible. Through ERCP, irrigation and aspiration of parasites in the biliary tract can aid in the removal of residual parasites and their eggs. It has the absolute advantage of minimal surgical trauma, less pain, and is conducive to recovery, which can prevent serious complications such as acute purulent obstructive cholangitis. It effectively alleviates jaundice and improves liver function, thereby enhancing the patient’s tolerance to surgery and reducing the risk of surgical complications. Moreover, ERCP can be employed to manage biliary complications arising from various conditions through biliary drainage procedures.^[24]^ The direct extraction of adult worms during endoscopy can achieve both diagnostic and therapeutic objectives, complemented by subsequent pharmacological anthelmintic treatment, which has been previously effective with tetrachloroethylene, clonidine and betel nut decoctions. Later, praziquantel (15 mg/kg) alone was effective even in severely infected fasciolopsiasis and is now the first-line treatment.^[25]^

## 4. Conclusion

The case report describes a rare instance of biliary tract infection caused by *F buski*, with successful extraction of adult worms under ERCP. A retrospective review of *F buski* infections in the digestive system over the past decade has enhanced physicians’ understanding of parasitic infections in this system. It also underscores the significant role of minimally invasive treatment in improving patient prognosis.

## Acknowledgments

The authors thank the patient and his family for providing consent for publication of this report. The authors also thank the reviewers for their helpful comments on this study.

## Author contributions

**Conceptualization:** Hongping Li.

**Supervision:** Hongping Li

**Writing – review & editing:** Hongping Li, Youcai Lv.

**Writing – original draft:** Qiao Zhou, Yu Cao, Qiuling Zhao, Yating Wei, Yunlan Gu, Juanjuan Yang, Luxi Yang.
